# Utility of a primary care based transient ischaemic attack electronic decision support tool: a prospective sequential comparison

**DOI:** 10.1186/1471-2296-15-86

**Published:** 2014-05-06

**Authors:** Annemarei Ranta, Chwan-Fen Yang, Michael Funnell, Pietro Cariga, Catherine Murphy-Rahal, Naomi Cogger

**Affiliations:** 1 Department of Neurology, MidCentral Health, Private Bag 11036, Palmerston North 4442, New Zealand; 2 Dean’s Department, University of Otago, Wellington, New Zealand; 3 Department of Medicine, Waikato Hospital, Hamilton, New Zealand; 4 EpiCentre, Massey University, Palmerston North, New Zealand

**Keywords:** Health service delivery, Electronic decision support, Transient ischaemic attack, Stroke, Stroke care, Secondary prevention

## Abstract

**Background:**

Stroke is a major cause of death and disability worldwide. Reducing the incidence of stroke has the potential to not only improve health outcomes, but also lead to significant cost savings for health services. Transient ischaemic attacks (TIA) can herald an imminent stroke and following a TIA early initiation of best medical therapy significantly reduces the risk of subsequent stroke. To achieve time targets rapid access stroke specialist services have been promoted; however, a number of resource related barriers can impede specialist access and cause unnecessary time delays. Cross sector collaboration led to the development of a primary care based TIA/Stroke electronic decision support (EDS) tool. This study aimed to assess the impact of this tool on improving access and reducing management delays.

**Methods:**

This is a prospective before (2009) versus after (2011) study of the effect on process of care following the implementation of EDS assisted TIA management in primary care. All patients presenting with TIA to secondary services were included. Outcomes assessed were TIA Guideline adherence and patient safety.

**Results:**

Over the study period 266 patients presented for TIA assessment (130 in 2009 and 136 in 2011). Following EDS implementation the median delay to specialist assessment fell from 10 days in 2009 to three days in 2011 (HR 1.45; 95% CI 1.13-1.86; p = 0.001), the number of patients achieving optimal medical therapy within 24 hours rose from 43% to 57% (RR 1.33; 95% CI 1.02-1.71; p = 0.04), carotid and CT imaging were achieved significantly faster (HR 1.52 (1.02-2.26) p = 0.003 and HR 1.34 (1.16-1.78 p = 0.002) respectively), and there were no adverse events associated with EDS use.

**Conclusion:**

The availability of TIA/Stroke electronic decision support in the primary care setting was associated with reductions in management delays without compromising patient safety.

## Background

Stroke is the third most common cause of death worldwide, the most common cause of long term adult disability in developed countries and represents a major burden on society both in terms of human and health services costs [1,2]. Ischaemic stroke is caused by an interruption of blood flow to the brain. This is typically caused by a blood clot, or thrombus, lodged in and blocking flow through cerebral arteries. Transient ischaemic attacks (TIAs) often herald an imminent disabling or fatal stroke [3-5] and early investigation and initiation of secondary prevention via rapid access specialist clinics has been shown to substantially reduce this risk [3,6,7]. However, most TIA and stroke studies come from tertiary university centres and many areas around the world struggle to mimic service models as proposed by the SOS-TIA [7] or EXPRESS [6] trials due to a variety of resource limitations.

In New Zealand, a low population density country, many rural and smaller urban areas lack the requisite patient volumes and health resources to make rapid (<24 hours) access outpatient specialist TIA clinics a feasible option and admitting all potential TIA patients to the hospital is too costly and often inappropriate due to a high rate of TIA mimics [8]. Other barriers to specialist access include geographical distances, patient financial constraints, as well as patient preference to be managed by their family doctor. In settings such as these innovative alternative models of care require exploration to achieve similar patient outcomes. Shifting some of the responsibility of caring for these patients back into the primary care sector is attractive from an access perspective, however, general practitioner (GPs) see TIA patients infrequently and may lack the confidence to initiate management without expert input [9]. The utilisation of new technologies such as computerised decision support tools offer an opportunity to provide generalists with additional support that goes beyond referencing a guideline. These tools are gaining in popularity throughout the health sector not only to improve access, but also to improve overall quality of care and cost-effectiveness [10].

To address the challenge of limited specialist access in rural and provincial New Zealand MidCentral Health neurologists collaborated with the primary care driven *Best Practice Advocacy Centre* (bpac) to design a TIA/Stroke Electronic Decision Support (EDS) tool intended to improve general practitioners’ (GPs) diagnostic accuracy, limit emergency department referrals to high risk patients, and prompt GPs to initiate secondary prevention immediately if specialist review is anticipated to be delayed or not achievable.

To ensure that the tool sufficiently mimics expert advice we conducted a study comparing expert, generalist, and software management of a number of hypothetical cases which demonstrated a high concordance rate between majority stroke expert opinion and software advice [9]. In addition, prior to launching the tool we conducted an eight week pilot [11] and subsequent to the launch a 14-month safety audit [12], both of which demonstrated high user satisfaction and no patient safety concerns. The aim of this study was to assess if the implementation of a TIA/Stroke EDS in the primary care setting would be associated with a reduction of avoidable TIA management delays without incurring additional patient risk.

## Methods

### Intervention

**Figure 1:**
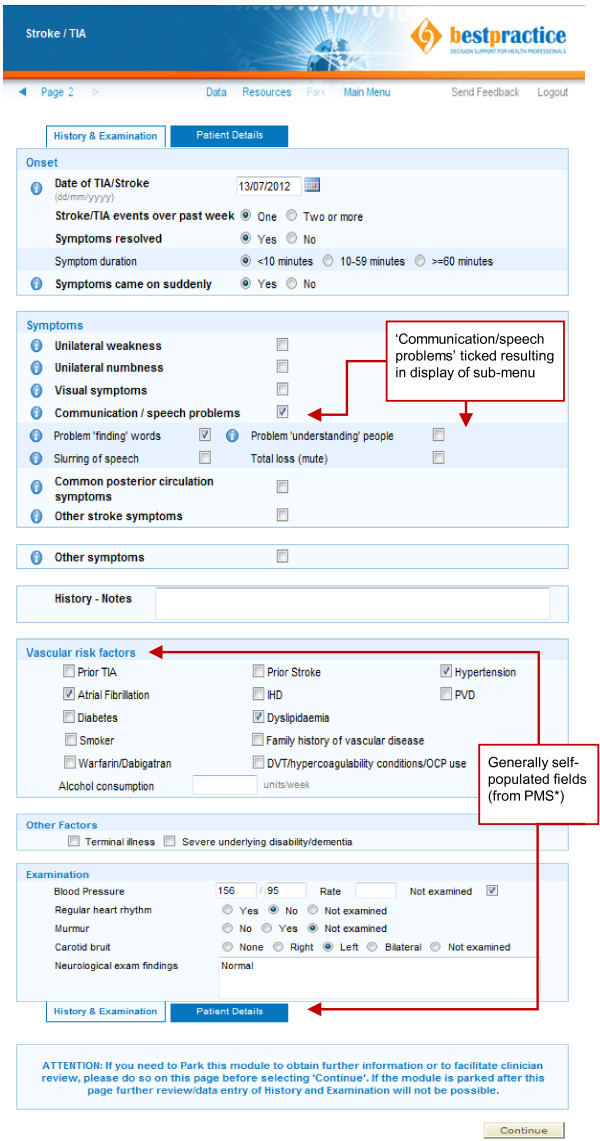
**TIA/Stroke electronic decision support data entry form depicting a sample case.** *PMS = Practice Management System i.e. GP electronic patient records.

**Figure 2:**
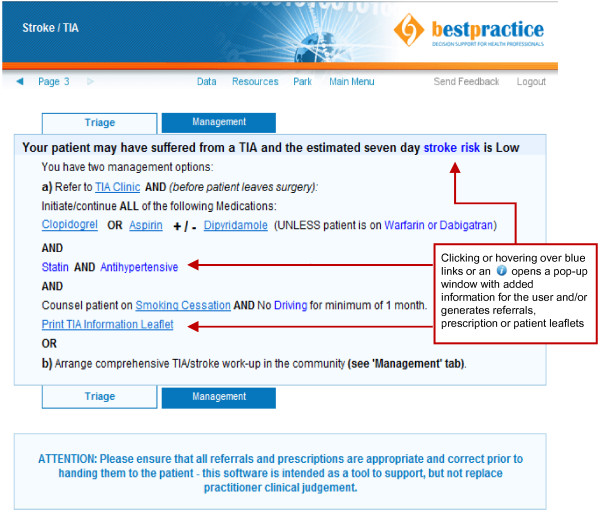
TIA/Stroke electronic decision support sample outcome page for a low risk patient with typical TIA symptoms.

The EDS tool consists of a web based single page data entry form that is completed by the GP (Figure 1). The computer algorithm incorporates diagnostic criteria and risk stratification in accordance with the New Zealand TIA guideline [13] as well as expert clinician experience. Unlike most electronic pathways the algorithm does not simply follow a vertical electronic flow-chart, but instead considers and carefully weighs specific clinical information provided concurrently to render a decision. This process aims to more closely mimic the decision making process of an experienced clinician, which is, in the case of TIA and many other medical conditions, considerably more complex than following a flow chart. For example, if three symptoms are listed one of which is typical for TIA and two are not clinical judgement has to be applied to weigh each individual symptoms in order to decide whether there is enough evidence to make TIA a likely diagnosis or not. In general, the computer algorithm errs on the side of over- rather than under-diagnosing TIAs to minimise risk and in the rare instance where a patient has no typical symptoms, but is still classed as ‘high risk’ applying the above criteria the tool still recommends urgent management. When a diagnosis of TIA or stroke is deemed likely a management recommendation is rendered based on risk stratification (Figure 2). A simplified diagnostic algorithm is shown in Additional file 1: Figure S1 and additional screenshots showing possible outcome pages can be viewed in prior publications [14].

Several strategies are in place to encourage GP utilisation of the tool. Firstly, the tool fully integrates into the GP electronic medical record system. This allows automatic population of a number of data entry fields with information previously documented (e.g. past medical history of atrial fibrillation or diabetes and demographic data). This avoids duplication and facilitates rapid data entry. Furthermore, the tool automatically generates specialist and investigation referrals, pre-populates prescriptions, prints tailored patient information leaflets, and provides links to additional educational materials. The tool is also inherently educational by providing the GP with definitions for neurological symptoms, guiding them into obtaining a focussed history and examination, and providing them with immediate diagnostic feedback. Lastly, by using the tool the GP may choose to manage the patient entirely on their own in the community. If the GP chooses to self manage and the tool supports a diagnosis of TIA the GP has rapid (<48 hour) access to head computed tomography (CT) and carotid ultrasound (if anatomic localisation supports a carotid territory TIA). Head CT access is otherwise generally limited to secondary care physicians in New Zealand and therefore the use of the tool offers GPs more autonomy when required. All of these features have been listed as favourable by surveyed GPs contributing to general end user uptake [11].

### Setting, design, and participants

The TIA/Stroke EDS was assessed in the MidCentral District Health Board (MDHB) after its launch in late 2009. MDHB provides public health services to approximately 170,000 people on New Zealand’s central North Island. In New Zealand’s public health system all hospital services, including investigations, are publicly funded and free of charge to patients. GP visits and prescription payments are subsidised, but require a co-payment. Some private specialist services are available although there were no private stroke service or neurology providers in the MDHB area during the study periods.

From January 2009 onward we prospectively identified all patients referred with a diagnosis of ‘TIA’ to the MidCentral outpatient TIA Clinic or the inpatient Acute Stroke Service. The study periods were from 1 January 2009 to 30 June 2009 (‘before’ EDS launch) and from 1 January 2011 to 30 June 2011 (‘after’ EDS launch) with 90 day follow-up periods.

Patients who were managed using the EDS, whether referred to secondary services or not, were independently identified via bpac’s central database and in addition underwent detailed primary care record review to screen for any adverse events that could potentially be linked to EDS use.

The New Zealand National Ethics Committee was consulted on the project and exempted the study from a formal ethics review because they considered this project an audit of an institutional service change.

### Study outcomes

There were four binary outcomes: 1) achievement of initiation of best medical therapy (BMT) within 24 hours of first presentation to a doctor; 2) documentation of behavioural counseling (including smoking cessation, diet/exercise, and driving advice); 3) achievement of CT scan; 4) achievement of carotid imaging. In addition there were three time-to event outcomes: 1) time to specialist, 2) time to brain computed tomography (CT), and 3) time to carotid imaging.

BMT looked at achievement of either anticoagulation for patients with atrial fibrillation (AF) or implementation of the combination of: 1) an antiplatelet or antiplatelet combination (Aspirin monotherapy was considered acceptable); 2) a statin, and 3) an anti-hypertensive. If a particular contraindication was listed the drug class was not required to achieve BMT. Patients who were already on BMT at the time of presentation were considered to have achieved BMT within 24 hours.

In addition, EDS safety was assessed by GP and hospital record review for any GP or hospital presentations that could possibly relate to the use of the EDS tool (e.g. due to a medication initiated based on EDS advice, or a mis-diagnosis or incorrect triage advice rendered by the EDS).

### Data analysis

All categorical data are presented as counts and percentages stratified by study period (2009/2011) and relative risks were calculated. The significance of association was assessed using the Chi-squared or the Fisher exact when the expected or actual cell count was less than five. Standard methods of survival analysis were used to investigate time to event outcomes. The unconditional association between this measure and the time period was assessed using the log-rank statistic. Multi-variate modeling was performed to assess for the impact of differences in baseline characteristics (smoking and ischaemic heart disease). All statistical tests were performed using R 2.15.

## Results

### Efficacy

In total 236 patients were included in the study: 130 in 2009 and 136 in 2011. All patients carried a referral diagnosis of ‘TIA’ and were eventually reviewed by a specialist who rendered a final diagnosis. Table 1 describes the baseline characteristics of patients at the initial presentation. A greater percentage of patients had ischaemic heart disease in 2009 (47% vs 29% ; p = 0.001) and a greater number of patients had a history of tobacco use in 2011 (22% vs 49%; p < 0.001). However, including these parameters in multi-variate modelling did not impact the significance of the following results.

**Table 1:** Baseline characteristics in patients who presented to the MidCentral Stroke Service before (2009) and after (2011) the introduction of a TIA/Stroke decision support tool

**Variable**	**2009**	**2011**	
	**(n = 130)**	**(n = 136)**	**p-value**
Male gender	57 (44)	69 (51)	0.26
Hypertension	88 (69)^*^	90 (66)	0.68
Diabetes mellitus	23 (18)^†^	18 (13)	0.34
Atrial fibrillation	34 (27) ^†^	29 (21)	0.28
Ischaemic heart disease	61 (48) ^†^	39 (29)	0.002
Dyslipidaemia	82 (65) ^†^	74 (54)	0.34
Current or ex-smoker	28 (29)^‡^	67 (56)^§^	0.0002
Stroke risk classified as high^f^	88 (68)	103 (76)	0.19
Age (years)			0.69
<60	26 (20)	33 (24)	
60-79	68 (52)	66 (49)	
>=80	36 (28)	37 (27)	
Best medical therapy at initial presentation	47 (36)	43 (32)	0.51

Data are number and percentage. Data missing for ^*^ 3, ^†^ 4, ^‡^ 35, and ^§^11 patients. ^f^Patients are classed as ‘high risk’ if they have ongoing stroke symptoms, an ABCD2 score ≥4, ≥2 events over preceding 7 days, atrial fibrillation, and/or receive anticoagulation therapy.

**Figure 3:**
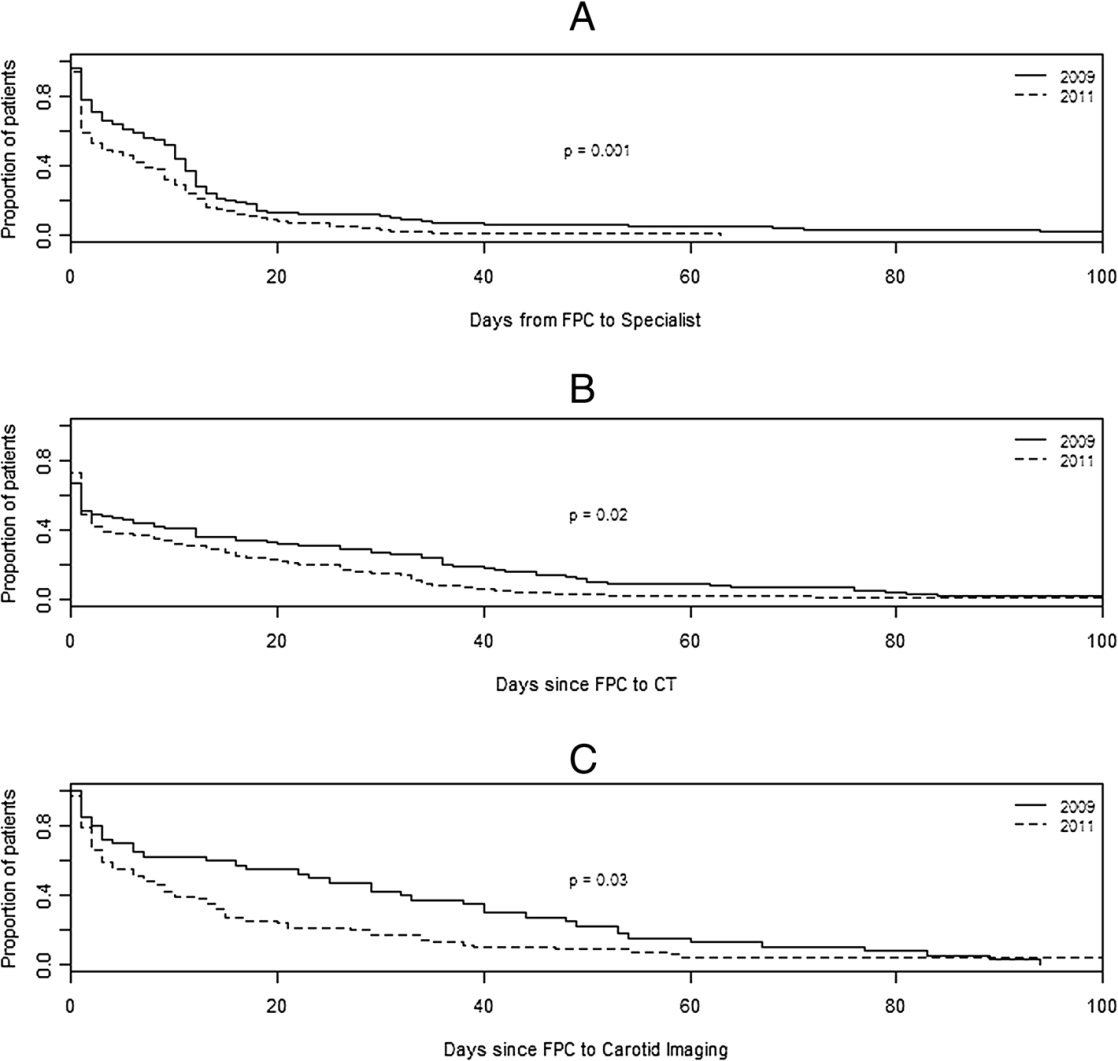
Kaplan-Meier estimate of the days from first point of contact (FPC) to review by a specialist (A), CT imaging (B), and carotid imaging (C) before (2009) and after (2011) the introduction of a TIA/Stroke electronic decision support tool.

Best medical therapy was achieved by 43% of patients in 2009 and 57% in 2011 (Relative Risk (RR) 1.33; 95% Confidence Interval (CI) 1.02-1.7; p = 0.04) and behavioural counseling was provided to 40% of patients in 2009 and 66% of patients in 2011 (RR 1.68; 95% CI 1.31-2.16; p < 0.0001) (Table 2).The time from first point of contact (FPC) until stroke specialist review was significantly shorter in 2011 than 2009 (p = 0.001; Figure 3). In 2011 the median time from FPC to specialist was 10 days which decreased to three days in 2011 (Hazard ratio (HR) 1.45; 95% CI 1.13-1.86).

**Table 2:** Primary outcomes before (2009) and after (2011) the introduction of a TIA/Stroke decision support tool

**Variable**	**2009**	**2011**	**Relative risk**	**P-value**
	**(n = 130)**	**(n = 136)**	**(95% CI)**	
BMT within 24 hours	51 (43)^†^	71 (57) ^§^	1.33 (1.02 - 1.71)	0.04
Behavioural counselling	51 (40)^*^	77 (66)^‡^	1.68 (1.31 - 2.16)	<0.0001
CT scan	93 (72)	117 (86)	1.3 (1.07 - 1.59)	0.006
Carotid imaging	40 (31)	71 (52)	1.7 (1.25 - 2.3)	0.0006

Data are number and percentage. Data missing for ^†^11, ^§^6, ^*^1, and ^‡^20 patients.

Patients seen in 2011 were 1.3 times (95% CI 1.07 - 1.59) more likely to have a CT scan than those seen in 2009 (p = 0.006; Table 1) and the median time till a CT scan was performed reduced from 2 days in 2009 to 1 day in 2011 (HR 1.34; 95% CI 1.16-1.78; p = 0.002; Figure 3). Similarly patients seen in 2011 were 1.7 (95% CI 1.25 - 2.3) more likely than patients seen in 2009 to have carotid imaging (p = 0.0006) and time to carotid imaging reduced from 24 days in 2009 to 7 days in 2011 (HR 1.52; 95% CI 1.02-2.26; p-value = 0.003; Figure 3).

### Safety

During the 2011 study period, five patients represented to hospital within 90 days of receiving EDS assisted management. Admission diagnoses included: hip fracture due to falls attributed to postural hypotension (unrelated to recent medication initiation), elective management of a patent foramen ovale (without recurrent TIA/stroke), bronchitis, astrocytoma, and migraine. While some of these diagnoses were related to the patients’ original ‘TIA’ presentation in no instance were these admissions adversely related to misdiagnosis or inappropriate management recommendations by the TIA EDS. There were no instances of even minor medication related adverse events or treatment delays due to EDS misdiagnosis or inappropriate triage advice. There were no cases of recurrent TIA or stroke amongst patients triaged as ‘low risk’ while awaiting outpatient specialist review. Similarly patients diagnosed as ‘non-TIA’ by the tool did not present with any subsequent cerebrovascular events.

## Discussion

We describe the first application of an electronic decision support tool in the primary care setting to aid in the management of TIA and minor strokes. This ‘Before and After’ study suggests that the implementation of this tool was associated with a significant improvement in the rate of rapid initiation of best medical TIA therapy, which has previously been shown to significantly reduce recurrent stroke [8]. In addition to earlier medication initiation we also observed a reduction in time delays to specialist review and relevant imaging. Furthermore, behavioural counseling and overall rate of diagnostic imaging acquisition improved after the tool was implemented.

Our data also suggest that this tool is safe. There were no cases of inappropriate diagnosis, triage, or management advice resulting in adverse events. Patients diagnosed as ‘non-TIAs’ did not experience any later recurrent TIAs or strokes and patients triaged as ‘low risk’ did not experience any recurrent TIAs or strokes while awaiting specialist review.

This study has clear limitations given its non-randomised observational design. While our comparison groups were identified prospectively and included all patients presenting to stroke specialists in the entire study population there remains a potential that some patients were missed because they were never referred to secondary care. There were also some baseline differences between groups: there was a higher prevalence of ischaemic heart disease in the 2009 and tobacco use in 2011. However, results remained significant even after adjusting for these potential confounders. As regards other potential service related confounders it is important to note that there were no changes relating to referral processing, admission criteria, appointment booking, diagnostic access, or neurology and radiology staff numbers between the two study periods. Nonetheless, despite our efforts to control for any changes other than the introduction of the TIA/Stroke EDS between study periods unrecognised confounders cannot, of course, be excluded in this type of study. For example, the promotion of the tool itself and wide dissemination of national TIA best practice guideline may have progressively raised TIA awareness throughout the district resulting in a general improvement in TIA care over time. A final limitation is that this study does not provide information about the impact of the tool on actual patient outcomes such as 90-day stroke risk. Complete 90-day follow-up data was not available for this sample and the study was not adequately powered to assess the tool’s impact on stroke recurrence. However, results from a multi-centre cluster randomised controlled trial, specifically designed to assess the tool’s impact on patient outcomes, will be available later this year and will provide further information [14].

## Conclusion

This cross-sectoral collaborative implementation of a TIA/Stroke EDS was associated with an improvement in TIA guideline adherence and a reduction in avoidable management delays, which have previously been linked to improved stroke outcomes and reduced health care costs. While our study design precludes us from asserting a clear causative link the findings nonetheless suggest that this type of health service provision may represent a feasible option to improve primary/secondary integration and improve overall TIA management and stroke prevention especially. This model would be particularly applicable in areas where more traditional models of care are difficult to replicate due to health resource limitations or geographical/cultural barriers impeding rapid specialist access. Results from a more definitive randomised controlled trial testing the efficacy and safety of this tool will be forthcoming later this year (FASTEST Trial ACTRN12611000792921).

## Abbreviations

TIA: Transient ischaemic attack; EDS: Electronic decision support; GP: General practitioner; BMT: Best medical therapy; FPC: First point of contact (with a doctor); CT: Computed tomography; MDHB: MidCentral District Health Board; RR: Relative risk; CI: Confidence interval; HR: Hazard ratio.

## Competing interests

The authors declare that they have no competing interests.

## Authors’ contributions

AR developed the TIA/Stroke EDS, designed the study, had overall oversight of study and final analysis/interpretation of data, and drafted the manuscript. CY led efficacy outcome data collection, participated in efficacy data analysis, and contributed to manuscript preparation. MF led safety data collection, analysed safety data, and contributed to manuscript preparation. PC contributed to study conception and design, identified study patients for data collection, and assisted with manuscript preparation. CM assisted with data collection and manuscript preparation. NC assisted with study design, performed statistical analysis, and assisted with manuscript preparation. All authors read and approved the final manuscript.

## Pre-publication history

The pre-publication history for this paper can be accessed here:

http://www.biomedcentral.com/1471-2296/15/86/prepub

## Supplementary Material

Additional file 1: Figure S1 Simplified TIA/stroke electronic decision support TIA diagnostic algorithm (excludes stroke and several more complex diagnostic features) [15]. Click here for file
