# Supplementary material for: Utility of a primary care based transient ischaemic attack electronic decision support tool: a prospective sequential comparison
Source: BMC Fam Pract. 2014 May 6;15:86. doi: 10.1186/1471-2296-15-86 (PMC4070650; doi:10.1186/1471-2296-15-86)
Supplement: Additional file 1: Figure S1 — Simplified TIA/stroke electronic decision support TIA diagnostic algorithm (excludes stroke and several more complex diagnostic features) [15]. [file 1471-2296-15-86-S1.docx]

“Non-straight forward neurological presentation” *

High Risk:

ABCD2^15^>3, atrial fibrillation, on anticoagulation, or one or more events over past 7 days

YES

NO

No Atypical Features

Any Atypical Features (non-sudden onset and/or atypical symptoms)

Typical TIA symptoms?

Low Risk:

ABCD2^15^<4 or event >7 days ago

Typical Features:

Unilateral weakness including facial droop

Unilateral numbness

Unilateral vision loss/Amurosis Fugax

Visual field loss/Hemianopia/Quadrantinopia

Binocular Dipolopia (‘double vision’)

Dysarthria (‘slurred speech’)

Dysphagia

Dysphasia (‘problems finding words’/’problems

understanding words’)

Dyspraxia/’clumsy hand’

Anosognosia/ hemi-neglect

Atypical Features:

Onset: Not sudden

Symptoms of:

Visual scotoma

Unilateral visual obscuration

Seizure

Syncope/Light-headedness/Pre-syncope

Acute memory Loss

Severe Headache

Bilaterally blurred vision

Agitation/Confusion/Inappropriate Behaviour/Apathy

Isolated vertigo/incoordination/loss of balance/ataxia

Somnolence

Other

TIA High Risk without community work-up override option

List atypical features

TIA High Risk with community work-up override option

TIA Low Risk with community work-up option

TIA Low Risk without community work-up option and list atypical symptoms

“Some symptoms not consistent with TIA”

List Atypical Features

TIA work-up option but

no community work-up option

With unilateral weakness

Without unilateral weakness

Atypical Features

No Atypical Features
